# Circulating anti‐p16a IgG autoantibodies as a potential prognostic biomarker for non‐small cell lung cancer

**DOI:** 10.1002/2211-5463.12535

**Published:** 2018-10-18

**Authors:** Huan Zhao, Xuan Zhang, Zhifeng Han, Yanjun Wang

**Affiliations:** ^1^ Second Hospital of Jilin University Changchun China; ^2^ Department of Thoracic Surgery China‐Japan Union Hospital Jilin University Changchun China

**Keywords:** autoantibody, NSCLC, p16, tumor immunity

## Abstract

It has been reported that p16 protein is overexpressed in many types of solid cancer and its aberrant expression may trigger the immune response, leading to the secretion of anti‐p16 antibodies. Here, we developed an in‐house ELISA with three p16‐derived linear peptide antigens to examine plasma anti‐p16 antibody levels in patients with non‐small cell lung cancer (NSCLC). Blood samples were taken from 200 control subjects and 211 patients with NSCLC prior to anticancer therapy. A Mann–Whitney *U* test demonstrated that plasma anti‐p16a IgG levels were significantly higher in NSCLC patients than in control subjects (*Z *=* *−11.14, *P *<* *0.001). However, neither plasma anti‐p16b nor plasma anti‐p16c IgG levels showed significant differences in patients with NSCLC as compared to control subjects. Moreover, further analysis indicated that anti‐p16a IgG levels increased with tumor stages, and patients with late stage NSCLC, namely group IV, had the highest IgG levels among four subgroups. Receiver operating characteristic analysis revealed that the anti‐p16a IgG assay had a sensitivity of 32.7% against a specificity of 95.0% in group IV, while Kaplan–Meier survival analysis revealed no significant difference in overall survival between patients with high anti‐p16a IgG levels and those with low anti‐p16a IgG levels (χ^2^ **=* *0.24, *P *=* *0.63). In conclusion, anti‐p16a IgG may be suitable for use as a prognostic biomarker for NSCLC.

**Abbreviations**
AC: adenocarcinoma
AUC: area under the ROC curve
CV: coefficient of variation
NSCLC: non‐small cell lung cancer
OS: overall survival
ROC: receiver operating characteristic
SBR: specific binding ratio
SCC: squamous cell carcinoma
TAA: tumor‐associated antigen

Cyclin‐dependent kinase inhibitor 2A, also called p16, is a negative regulator of the cell cycle in the late G1 phase 1, and could arrest cell cycle progression from G1 to S phase by inhibiting cyclin‐dependent kinase 4‐ and cyclin‐dependent kinase 6‐mediated phosphorylation of retinoblastoma (Rb) protein 2. p16^Ink4a^ gene mutation has widely been reported in nearly 50% of human cancers 3. Point mutation, promoter methylation and homozygous deletions of chromosome 9p21 could all lead to inactivation of the p16^Ink4a^ gene, which may be an early and critical event in cancer development and progression 4, 5. A number of studies have demonstrated that overexpression of p16 was often observed in a wide spectrum of cancer types and a progressive increase of p16 protein was associated with malignant transformation of tumors 3.

Several lines of evidence have suggested that the humoral immune response to tumor‐associated antigens (TAAs) is developed rapidly in the initiation and development of tumors 6, 7. If the autoantibody to p16 protein can be detected before symptomatic disease 8, it could be a useful biomarker to detect cancer at a curable stage. Although circulating anti‐p16 antibodies have been found to be increased in several types of solid tumors, such as nasopharyngeal cancer 9, hepatocellular carcinoma 9, breast cancer 10, esophageal cancer 11, cervical cancer 12 and non‐small cell lung cancer (NSCLC) 13, the reports to date on altered anti‐p16 antibody levels in early stage cancer remain inconsistent. Accordingly, the present study was designed to develop an ELISA in‐house to explore how plasma anti‐p16 IgG levels were changed in patients with NSCLC and whether anti‐p16 IgG could be used as diagnostic biomarkers for screening of NSCLC.

## Materials and methods

### Subjects

A total of 211 patients, who were first diagnosed with NSCLC at the Department of Thoracic Surgery, China‐Japan Union Hospital of Jilin University in the period between November 2012 and August 2016, were recruited in this study; demographic details and clinical characteristics are given in Table 1. Blood sample was collected from each patient prior to any anticancer treatment received. This study was restricted to squamous cell carcinoma (SCC) and adenocarcinoma (AC) only. Clinical stages of NSCLC were determined based on the TNM staging system. To clarify how plasma anti‐p16 IgG levels were changed in patients with different stages of NSCLC, we divided all patients into four subgroups: group I for stage T_1_N_0_M_0_, group II for stage T_1_N_1_M_0_ + T_2_N_0_M_0_, group III for stage T_2_N_1_M_0_ + T_3_N_0_M_0_ and group IV for stages 3 and 4. Control subjects were recruited from local communities during the same period as sample collection from patients, and 200 eligible healthy subjects who had no history of cancer or autoimmune diseases were selected for this study. All patients were followed up until death or censoring, and clinical follow‐up data were obtained from the Large‐scale Data Analysis Center of Cancer Precision Medicine‐LinkDoc database 14.

**Table 1:** Demographic information and clinical characteristics of NSCLC patients

Characteristic	No. of patients	%
Age (years)		
≥ 60	106	50.2
< 60	105	49.8
Gender		
Male	131	62.1
Female	80	37.9
Smoking history		
Smoker	106	50.2
Nonsmoker	105	49.8
Histology		
Squamous cell carcinoma	87	41.2
Adenocarcinoma	124	58.8
TNM group^a^		
I	20	9.5
II	101	47.9
III	41	19.4
IV	49	23.2

a Group I for stage T_1_N_0_M_0_, group II for stage T_1_N_1_M_0_ + T_2_N_0_M_0_, group III for stage T_2_N_1_M_0_ + T_3_N_0_M_0_ and group IV for stages 3 and 4.

Written informed consent to take part in this study and donate blood samples was obtained from all participants. This study was approved by the Ethics Committee of Second Hospital of Jilin University and conformed to the Declaration of Helsinki.

### Detection of plasma IgG levels

Three linear peptide antigens derived from p16 protein, namely p16a, p16b and p16c, were designed using computational epitope prediction software (http://www.iedb.org) and synthesized by solid‐phase chemistry with a purity of > 95% (Table 2). An in‐house ELISA was then developed with these linear peptides as described in previous reports 15, 16, 17. All assays were performed in duplicate, and the specific binding ratio (SBR) was used to represent plasma anti‐p16 IgG levels. SBR is calculated as follows (where NC is negative control and PC is positive control):

$$\text{SBR}=\left(A_{\mathrm{sample}}-A_{\mathrm{NC}}\right)/\left(A_{\mathrm{PC}}-A_{\mathrm{NC}}\right)$$

**Table 2:** Information for peptide antigens derived from p16

Antigen	Sequence (N→C)	NCBIA accession	Position (aa)
p16a	CGFLDTLVVLHRAGARLDVRDAWGR	NP_000068	89–102
p16b	CDLAEELGHRDVARYLRAAAGGTRGS	NP_000068	116–140
p16c	GTRGSNHARIDAAEGPSEMIGNHLWVC	NP_000068	136–162

The inter‐assay deviation of the in‐house ELISA was estimated by a quality control sample that was pooled from > 100 plasma samples randomly taken from unrelated healthy individuals; the coefficient of variation (CV) was used to represent reproducibility of the in‐house ELISA.

Total IgG levels in plasma were measured by IgG (Total) Human Uncoated ELISA Kit with Plates (Cat. 88‐50550, Thermo Scientific, Shanghai, China) based on manufacturer's instruction.

### Data analysis

Normality of the distribution of plasma IgG levels was tested by the Kolmogorov–Smirnov one‐sample test (Table 3). Due to the skewed distribution of plasma anti‐p16 IgG levels in control subjects, a Mann–Whitney *U* test was performed for comparison of anti‐p16 IgG levels between the patient group and the control group; Student's *t*‐test was used to examine the difference in anti‐p16 IgG levels between group I and the combination of other three subgroups. Receiver operating characteristic (ROC) curve analysis was applied to work out the area under the ROC curve (AUC) with 95% confidence interval (CI), and the sensitivity of ELISA antibody test against a specificity of ≥ 95%.

**Table 3:** Kolmogorov–Smirnov test for a normal distribution of plasma IgG levels

Antibody	Skewness	Kurtosis	*P*
p16a			
Patient	0.152	−0.288	0.2
Control	0.568	0.193	0.007
p16b			
Patient	1.824	6.606	< 0.001
Control	1.133	3.11	0.011
p16c			
Patient	0.807	0.605	< 0.001
Control	0.563	1.0	0.001
Total IgG			
Patient	0.193	−0.326	0.2
Control	0.225	−0.414	0.028

Patients were also dichotomized as low IgG level subgroup and high IgG level subgroup based on the medians of plasma IgG measurements. The overall survival (OS) was defined as the period between the date of first diagnosis and that of death or censoring. If the cumulative survival rate did not reach below 50%, the mean ± standard error (SE) was used to represent the OS of patients with NSCLC. Kaplan–Meier survival analysis and Cox regression were performed to examine the difference in OS between the two subgroups classified based on plasma anti‐p16 IgG levels.

## Results

The in‐house ELISA showed a good reproducibility with a CV of 9.8% for the anti‐p16a IgG assay, 14.4% for the anti‐p16b IgG assay and 11.2% for the anti‐p16c IgG assay.

The Mann–Whitney *U* test showed that plasma anti‐p16a IgG levels were significantly higher in NSCLC patients than in control subjects (*Z *=* *−11.14, *P *< 0.001), both male and female patients contributing to the increased anti‐p16a IgG levels (*Z *= −9.11, *P *<0.001 in males and *Z *= −6.05, *P *<* *0.001 in females). However, there was no significant difference in plasma IgG levels for p16b and p16c between NSCLC patients and control subjects (Table 4).When all subjects were divided into two subgroups based on their ages, namely group 1 aged ≥ 60 years old and group 2 aged < 60 years old, both the two age groups showed higher anti‐p16a IgG levels than the control group (Table 5); both SCC and AC contributed to increased anti‐p16a IgG levels (Table 6). Further analysis showed that plasma anti‐p16a IgG levels were increased in all four stage subgroups, and group IV had the highest anti‐p16a IgG levels (Table 7) although Student's *t*‐test failed to show a significant difference in anti‐p16a IgG levels between group I and the combination of the other three subgroups (*t *=* *1.61, df* *=* *210, *P *=* *0.109).

**Table 4:** Analysis of plasma anti‐p16 IgG levels in patients with NSCLC and control subjects. The antibody levels are expressed as mean ± SD in SBR. Values of *Z* are calculated from a Mann–Whitney *U* test (two‐tailed)

IgG	Group	Patient (*n*)	Control (*n*)	*Z*	*P*
P16a	Male	0.77 ± 0.16 (131)	0.53 ± 0.16 (103)	−9.11	< 0.001
	Female	0.72 ± 0.20 (80)	0.53 ± 0.17 (97)	−6.05	< 0.001
	Both	0.75 ± 0.18 (211)	0.53 ± 0.17 (200)	−11.14	< 0.001
P16b	Male	0.89 ± 0.29 (131)	0.83 ± 0.25 (103)	−1.65	0.1
	Female	0.88 ± 0.35 (80)	0.92 ± 0.26 (97)	−1.67	0.09
	Both	0.89 ± 0.31 (211)	0.87 ± 0.26 (200)	−0.04	0.97
P16c	Male	0.94 ± 0.25 (131)	0.86 ± 0.20 (103)	−1.91	0.06
	Female	0.94 ± 0.25 (80)	0.95 ± 0.23 (97)	−0.60	0.55
	Both	0.94 ± 0.25 (211)	0.90 ± 0.22 (200)	−0.96	0.34

**Table 5:** Analysis of plasma anti‐p16 IgG levels in different age groups. Plasma IgG levels are expressed as mean ± SD SBR. Values of *Z* are from a Mann–Whitney *U* test (two‐tailed). *P* < 0.0125 was considered to be statistically significant as four individual antigens were tested

IgG	Age (years)	Patient (*n*)	Control (*n*)	*Z*	*P*
p16a	≥ 60	0.77 ± 0.17 (106)	0.53 ± 0.18 (99)	−8.20	<0.001
	< 60	0.73 ± 0.19 (105)	0.52 ± 0.16 (101)	−7.43	<0.001
p16b	≥ 60	0.92 ± 0.35 (106)	0.86 ± 0.24 (99)	−0.62	0.54
	< 60	0.86 ± 0.27 (105)	0.88 ± 0.28 (101)	−0.53	0.60
p16c	≥ 60	0.96 ± 0.25 (106)	0.91 ± 0.22 (99)	−1.0	0.32
	< 60	0.92 ± 0.24 (105)	0.90 ± 0.22 (101)	−0.31	0.76

**Table 6:** Analysis of plasma anti‐p16 IgG levels in two histological types of NSCLC. The antibody levels are expressed as mean ± SD SBR. Values of *Z* are from a Mann–Whitney *U* test (two‐tailed). AC, adenocarcinoma; SCC, squamous cell cancer

IgG	Type	Patient (*n*)	Control (*n*)	*Z*	*P*
p16a	SCC	0.79 ± 0.17 (87)	0.53 ± 0.17 (200)	−9.58	< 0.001
	AC	0.72 ± 0.18 (124)	0.53 ± 0.17 (200)	−8.80	< 0.001
p16b	SCC	0.91 ± 0.33 (87)	0.87 ± 0.26 (200)	−0.63	0.53
	AC	0.87 ± 0.30 (124)	0.87 ± 0.26 (200)	−0.44	0.66
p16c	SCC	0.96 ± 0.26 (87)	0.90 ± 0.22 (200)	−1.40	0.16
	AC	0.92 ± 0.24 (124)	0.90 ± 0.22 (200)	−0.31	0.76

**Table 7:** Analysis of plasma anti‐p16 IgG levels in four subgroups of NSCLC stages. The antibody levels are expressed as mean ± SD SBR. Values of *Z* are from a Mann–Whitney *U* test (two‐tailed)

TAAs	Group	Patient (*n*)	Control (*n*)	*Z*	*P*
P16a	I	0.69 ± 0.23 (20)	0.53 ± 0.17 (200)	−2.93	0.003
	II	0.74 ± 0.18 (101)	0.53 ± 0.17 (200)	−8.7	< 0.001
	III	0.77 ± 0.14 (41)	0.53 ± 0.17 (200)	−7.48	< 0.001
	IV	0.78 ± 0.18 (49)	0.53 ± 0.17 (200)	−7.44	< 0.001
p16b	I	0.85 ± 0.41 (20)	0.87 ± 0.26 (200)	−1.02	0.31
	II	0.86 ± 0.24 (101)	0.87 ± 0.26 (200)	−0.18	0.86
	III	0.93 ± 0.30 (41)	0.87 ± 0.26 (200)	−1.10	0.27
	IV	0.93 ± 0.41 (49)	0.87 ± 0.26 (200)	−0.01	0.99
p16c	I	0.90 ± 0.31 (20)	0.90 ± 0.22 (200)	−0.83	0.41
	II	0.93 ± 0.24 (101)	0.90 ± 0.22 (200)	−0.18	0.86
	III	0.98 ± 0.21 (41)	0.90 ± 0.22 (200)	−2.20	0.03
	IV	0.96 ± 0.27 (49)	0.90 ± 0.22 (200)	−0.79	0.43

**Figure 1:**
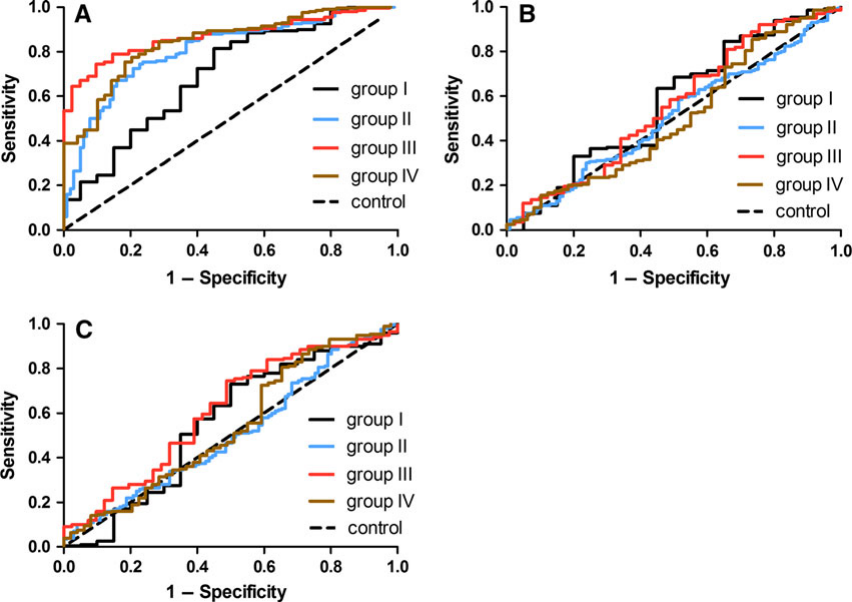
ROC curve analysis of plasma anti‐p16 IgG levels for four subgroups of NSCLC. (A) Plasma anti‐p16a IgG levels; (B) Plasma anti‐p16b IgG levels; (C) Plasma anti‐p16c IgG levels.

ROC curve analysis showed that the anti‐p16a IgG assay had an AUC of 0.818 (95% CI 0.777–0.859) with a sensitivity of 24.2% against the specificity of 95.0%, the anti‐p16b IgG assay had an AUC of 0.501 (95% CI 0.445–0.557) with a sensitivity of 7.1% against the specificity of 95.0%, and the anti‐p16c IgG assay had an AUC of 0.527 (95% CI 0.471–0.583) with a sensitivity of 9.0% against the specificity of 95.0% (Table 8; Fig. 1). There was no significant difference in total IgG levels between the patient group and the control group (3.00 ± 1.14 mg·mL^−1^ in the patient group and 3.10 ± 1.08 mg·mL^−1^ in the control group, *Z *=* *−0.73, *P *=* *0.46).

**Table 8:** ROC analysis of plasma anti‐p16 IgG levels in four subgroups of NSCLC stages. SE, standard error. Values of sensitivity are against a specificity of 95.0%

TAAs	Group	AUC	SE	95% CI	Sensitivity (%)
p16a	I	0.699	0.065	0.571–0.827	20.0
	II	0.807	0.026	0.756–0.858	21.8
	III	0.871	0.023	0.825–0.917	22.0
	IV	0.843	0.029	0.786–0.900	32.7
	Overall	0.818	0.021	0.777–0.859	24.2
p16b	I	0.569	0.075	0.423–0.715	15.0
	II	0.506	0.035	0.437–0.576	4.0
	III	0.555	0.053	0.451–0.658	12.2
	IV	0.501	0.049	0.404–0.597	12.2
	Overall	0.501	0.029	0.445–0.557	7.1
p16c	I	0.556	0.077	0.405–0.707	5.0
	II	0.506	0.036	0.436–0.576	7.9
	III	0.609	0.05	0.51–0.708	4.9
	IV	0.537	0.049	0.44–0.633	12.2
	Overall	0.527	0.029	0.471–0.583	9.0

**Figure 2:**
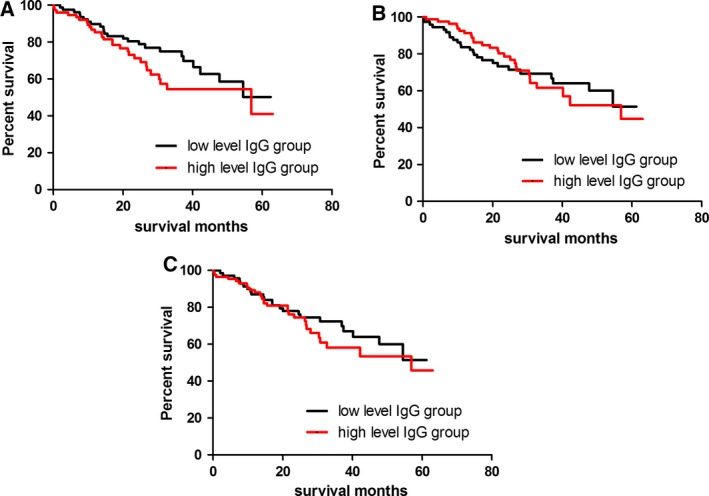
Kaplan–Meier survival analysis for plasma anti‐p16 IgG and OS in patients with NSCLC. (A) Plasma anti‐p16a IgG levels; (B) Plasma anti‐p16b IgG levels; (C) Plasma anti‐p16c IgG levels.

Of 154 patients who were successfully followed up, 52 died prior to the last follow‐up performed in December 2017. The Kaplan–Meier survival analysis and Cox regression showed no significant difference in OS between patients with high anti‐p16 IgG levels and those with low anti‐p16 IgG levels (Table 9; Fig. 2).

**Table 9:** Kaplan–Meier survival analysis of differences in overall survival between NSCLC patients with low IgG levels and those with high IgG levels. Values for overall survival are mean ± SE. χ^2^ was calculated from Cox regression analysis when anti‐p16 IgG levels were analyzed as continuous variables. *P*‐values are uncorrected for age, gender, NSCLC stages and types

IgG	Overall survival (months)		χ^2^	*P*
	Low‐level group	High‐level group		
p16a	47.6 ± 2.55	43.7 ± 2.98	0.24	0.63
p16b	46.3 ± 2.67	44.8 ± 2.79	1.14	0.29
p16c	46.6 ± 2.61	44.7 ± 2.84	1.94	0.16

## Discussion

The p16 protein is a well‐known tumor suppressor molecule, and its inactivation is likely to be associated with tumor development. Intriguingly, the overexpression of p16 protein has been reported in several types of solid tumors such as cervical cancer 12 and lung cancer 18. Several studies suggested that aberrant expression of p16 could start in an early stage of cancer development and was gradually increased with tumor progression 19, 20, 21. In our study, we found that plasma anti‐p16a IgG levels were progressively increased with tumor stages and NSCLC patients in a late stage (group IV) had the highest IgG levels among four subgroups (Table 7). Our findings were consistent with the report by Zhang *et al*. 13, but controversial with regard to the results reported by Jin and co‐workers who found that plasma anti‐p16a IgG levels were inversely correlated with stages of esophageal cancer and patients at stage I had the highest IgG levels 11. It is possible that the pattern of changes in anti‐p16 antibody levels varies between tumor types. It is worth noting that the anti‐p16a IgG assay showed a sensitivity of 32.7% against a specificity of 95.0% in group IV, raising the possibility that plasma anti‐p16a IgG may have a prognostic value for NSCLC, although there was no significant difference in OS between patients with high anti‐p16a IgG levels and those with low anti‐p16a IgG levels (Fig. 2; Table 9). Failure to show a significant correlation between anti‐p16 IgG levels and OS of NSCLC suggests that patients with a late stage NSCLC may have impaired immune responses to increased p16‐derived antigens due to chemotherapy or other unknown reasons.

Although the mechanism behind increased anti‐p16a IgG levels in NSCLC remains unclear, some evidence indicates that alterations of the p16^Ink4a^–Rb pathway may be involved in cancer development and progression 3, 22. Due to the existence of positive feedback, the loss of Rb protein may result in the overexpression of p16 23, although such an attempt to suppress the proliferation of tumor cells is fruitless 22. The inverse correlation between p16^Ink4a^ and Rb expression has been described in lung cancer 24, 25. However, the present work did not examine the alteration of Rb protein and this is a limitation of this study.

Taken together, the present study applied an in‐house ELISA for detection of plasma IgG antibodies against three p16‐derived peptide antigens. The results suggest that humoral immune responses to these antigenic peptides may be different in individual patients with NSCLC, and only p16a rather than p16b and p16c could stimulate humoral immune responses. In fact, each protein may carry many different epitopes that can be recognized by differential B cell receptors and in turn trigger different immune responses, leading to the production of diverse autoantibodies 26. If this is a case, mapping powerful epitopes will be helpful to develop more sensitive antibody tests for identification of TAA‐related biomarkers and therapeutic targets.

## Author contributions

HZ carried out experiments, performed the data analysis and drafted the manuscript; XZ conceived and designed this study. YW supervised all laboratory work and data analysis. ZH was mainly responsible for the collection of blood samples and clinical information. All authors have reviewed the manuscript.

## Conflict of interest

The authors declare no conflict of interest.
